# Clear Cell Ovarian Carcinoma With C1 Lateral Mass Metastasis and Pathologic Fracture: A Case Report

**DOI:** 10.7759/cureus.34766

**Published:** 2023-02-08

**Authors:** Dib Sassine, Daniella Rogerson, Matei Banu, Patrick Reid, Caryn St. Clair

**Affiliations:** 1 Gynecologic Oncology, Columbia University Department of Obstetrics and Gynecology, New York, USA; 2 Obstetrics and Gynecology, Columbia University Vagelos College of Physicians and Surgeons, New York, USA; 3 Neurosurgery, Columbia University Department of Neurological Surgery, New York, USA; 4 Neurosurgery, Columbia University, New York, USA

**Keywords:** ovarian clear cell carcinoma, vertebral fusion, pathological fracture, osseous metastasis, ovarian carcinoma

## Abstract

Osseous metastasis (OM) in ovarian cancer (OC) are rare, with an incidence ranging from 0.8% to 2.6%, and are associated with poor prognosis. The available literature on their management and associated complications is scarce.

We report a case of International Federation of Gynecology and Obstetrics (FIGO) stage IVB clear cell epithelial OC (EOC) who presented with neck pain. Imaging revealed multiple cervical spine metastases with left vertebral artery encasement and concurrent C1 lateral mass compression fracture, without neurological deficit, requiring occiput to C2 posterior instrumentation and fusion.

Early OM may be associated with shorter overall survival, and survival after OM diagnosis is on the order of months. Management of OM should include a multidisciplinary team and may require surgical stabilization in addition to systemic chemotherapy, local radiotherapy, and osteoclast inhibitors.

## Introduction

Ovarian cancer (OC) is the most common cause of gynecological cancer death [1]. Direct spread to adjacent pelvic organs, peritoneal spread, and metastasis via the lymphatic route are common, while hematogenous spread to further sites such as the liver, lung, bone and brain occur less commonly and have a poor prognosis [2-4]. Osseous metastases (OM) in OC are among the rarest, with incidences between 0.82% and 4.0%, and may occur by direct invasion, hematogenous, lymphatic, and transperitoneal spread [2, 3, 5, 6, 7]. The vertebral column is the most common site, though case reports document findings throughout the axial skeleton and pelvis [5]. OMs are associated with shorter overall survival [4]. A recent study found the 1-, 3- and 5-year survival after OM diagnosis were 33%, 15%, and 8%, respectively [8]. Skeletal-related events (SREs) and neurovascular compromise secondary to OM have rarely been described. Here, we report a case of International Federation of Gynecology and Obstetrics (FIGO) stage IVB clear cell epithelial ovarian cancer (EOC) complicated by C1 vertebral metastases with vertebral artery involvement and C1 vertebral fracture, necessitating occiput to C2 posterior instrumentation and fusion and palliative C2 neurectomy.

## Case presentation

A 59-year-old African American P0 (nulliparous) with a history of uterine leiomyoma presented with abdominal pain and bloating. A transabdominal sonogram demonstrated multiple large, complex adnexal masses. Staging computed tomography (CT) chest, abdomen and pelvis was suspicious for metastatic ovarian carcinoma, with bilateral complex adnexal masses, peritoneal carcinomatosis, omental caking, and pericardiac, abdominal, peripancreatic, mesenteric, retroperitoneal, and pelvic lymphadenopathy. No osseous lesions were identified at the time of the initial scan.

An initial consult with gynecological oncology revealed an abdominal mass above the umbilicus, pelvic masses, and a palpable left supraclavicular node. Ca-125 was 6,773. Pathological examination of interventional radiology-guided peritoneal mass biopsies had an immunoprofile compatible with a high-grade adenocarcinoma of Mullerian origin, favoring clear cell carcinoma. The tumor showed preserved mismatch repair (MMR) proteins expression (positive for MLH1, PMS2, MSH2, MSH6). Immunostain for human epidermal growth factor receptor 2 (HER-2) was equivocal and difficult to interpret on the scant cell block material with mostly cytoplasmic staining, no convincing membranous staining, score 0-1, and immunostaining for PD-L1 was limited by scant cellularity in the cell block, focal positivity with an estimated combined positive score (CPS) 3-4. Further genetic testing did not show any mutations for possible targeted therapy. The patient was diagnosed with FIGO stage IVB clear cell EOC. She received her first cycle of carboplatin, paclitaxel, and bevacizumab, with a plan for 3-4 cycles of neoadjuvant chemotherapy and interval debulking pending clinical response.

**Figure 1:**
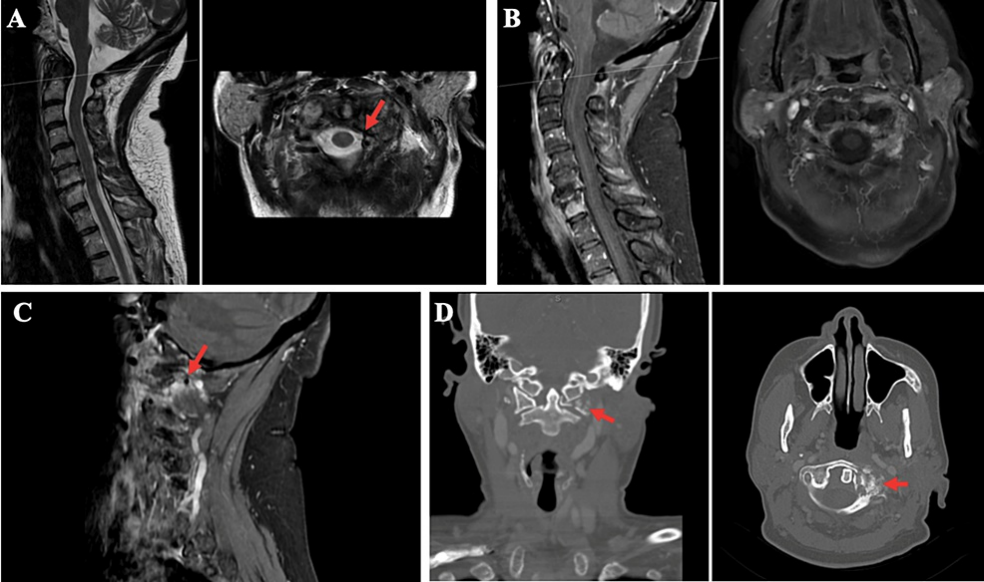
Sagittal (left) and axial (right) MRI of the patient A. T2-weighted and B. T1 fat saturation MRI sequences demonstrating C1 lateral mass lesion (red arrow) and displacement of the vertebral artery with no epidural tumor extension or cord compression. C. Parasagittal T1 sequence demonstrating lateral tumor extension with encasement of the vertebral artery (red arrow). D. Coronal (left) and axial (right) CT angiogram demonstrating osteolytic left lateral mass lesion encasing and narrowing the vertebral artery (red arrows).

After her first cycle of chemotherapy, the patient endorsed persistent neck pain of moderate severity. Magnetic resonance imaging (MRI) cervical spine revealed contrast-enhancing osseous lesions within the anterior arch and left lateral mass of C1, as well as in the C4 and C7 vertebral bodies, left articulating facet of C6 and T4 vertebral body extending into the left pedicle. There was lateral tumor extension with encasement of the vertebral artery at the C1 level. There was no evidence of epidural disease or spinal cord impingement (Figure 1A-1C). CT angiogram (CTA) demonstrated multiple osseous lytic lesions and a C1 lateral mass compression fracture extending to the left transverse foramen, with asymmetry of the lateral atlantodental interval measuring 8 mm on the right and 2 mm on the left. There was circumferential tumor encasement of the left vertebral artery in the sulcus arteriosus, with severe narrowing with preserved flow and no evidence of dissection (Figure 1D). Metastatic cervical and supraclavicular lymphadenopathy were confirmed. The total body bone scan did not show further OM.

The patient was admitted to neurosurgery with gynecological oncology consultation, where she continued to endorse left-sided cervical pain radiating to the occiput which worsened with movement and improved with opioids, steroids and immobilization with a hard collar. There were no focal neurological deficits, paresthesias, anesthesia, gait difficulties, or bowel or bladder dysfunction. The patient had full strength in the upper and lower extremities and an intact gait. She had no signs of myelopathy and had a negative Hoffman’s sign.

**Figure 2:**
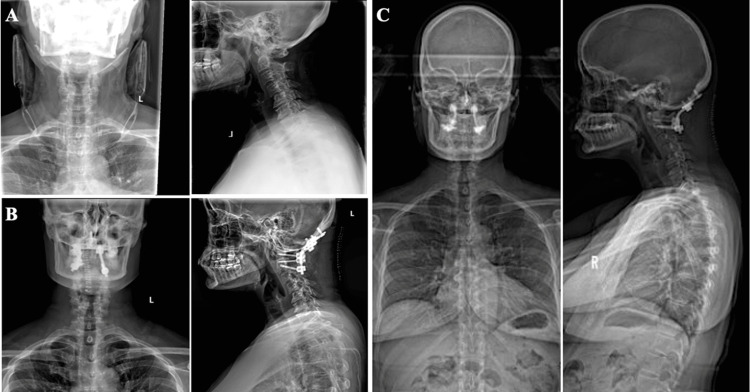
Preoperative (A) and postoperative (B and C) radiographs A. Preoperative anterior-posterior (left) and upright lateral (right) radiographs demonstrating normal cervical spine alignment. B. Postoperative anterior-posterior (left) and lateral (right) cervical spine radiographs demonstrating appropriate instrumentation placement and alignment after posterior hardware fixation of the occiput to C2. C. Postoperative anterior-posterior (left) and lateral (right) standing scoliosis series illustrating normal alignment.

Given normal cervical alignment, with imaging demonstrating articular facet involvement and lateral mass fracture, this pain was considered to be caused by cervical spine instability (Figure 2A). 

Urgent surgery was not indicated without neurological deficits or compressive pathology. A multidisciplinary team discussed the case to determine if surgical decompression was required prior to local radiation, and surgical intervention with occiput to C2 instrumentation and fusion was decided upon. Surgery was performed two days after diagnosis. Intraoperatively, there was extensive tumor involvement of the C1 lateral mass and posterior arch, encasing the vertebral artery and extending towards the occipital condyle. A palliative C2 neurectomy was performed for pain control. A right C1 lateral mass screw, bilateral C2 pedicle screws, and occipital keel plate with three bicortical screws were placed. The fusion bed was prepared by decorticating the occiput and bilateral C1-2 joint spaces with the placement of allograft over the decorticated spaces. Intraoperative monitoring was stable throughout and the patient awoke at neurological baseline. Postoperatively, the patient endorsed significant improvement of her cervical pain. Incisional pain was controlled with methocarbamol, gabapentin and hydromorphone PCA (patient-controlled analgesia). Radiographs demonstrated excellent instrumentation placement and alignment (Figure 2B).

The patient ambulated without assistance on postoperative day (POD) 1 and was discharged in stable condition on POD3. Radiographs of the spine confirmed stable spinal fusion rods three weeks postoperatively (Figure 2C). There was complete resolution of the cervical pain. The patient received her second cycle of carboplatin and paclitaxel four weeks postoperatively; at that time, progression of cervical lymphadenopathy was noted on exam. She since received two additional cycles three and six weeks later, with a downtrending CA-125 of 2,301. Bevacizumab has been held to allow for surgical healing. Denosumab injection of 120 mg every 4 weeks, calcium, and vitamin D were initiated postoperatively, with a plan for palliative radiation therapy (RT) of 20 Gy in 5 fractions to C1-2 and associated hardware to prevent disease progression.

## Discussion

This report illustrates a unique complication of OM in EOC. Given the rare nature of these lesions, the literature is sparse. Four retrospective studies comment on OM in the context of other rare OC metastases [4, 9, 10, 11]. In these, the incidence of OM ranged between 1.2% and 4%. Deng et al. [4] analyzed 1481 patients with stage 4 ovarian carcinoma using the Surveillance, Epidemiology, and End Results (SEER) database. OM was found in 3.74% of the entire cohort with a median survival time of 11 months for the patients with OM. Dauplat et al. [9] examined 255 patients with EOC with stage 4 disease. Only four patients (1.6%) had OM with a median survival of 9 months. Gardner et al. [10] used the SEER database to determine the pattern of the distant metastases at the initial presentation in patients with gynecological cancer and found that OM was present in 4% of the patients with ovarian carcinoma, vs 13% and 23% of patients with uterine and cervical cancer, respectively. 

Additionally, four retrospective studies looked specifically at OM in OC [2, 5, 6, 7] (Table 1).

**Table 1:** Four retrospective studies examine osseous metastasis (OM) in ovarian carcinoma

Publication – Author, Year	Cohort – No	OM – No (%)	Overall Survival – Months	Survival After OM Diagnosis – Months	Most Commonly Involved Site
Ak et al., 2021 (2)	736	19 (2.60)	38.1	13.6	Vertebral
Sehouli et al., 2013 (5)	1,717	26 (1.50)	50.5	7.2	Vertebral
Zhang, C. et al., 2019 (6)	32,178	352 (1.09)	50.0	5.0	Not Reported
Zhang, M. et al., 2013 (7)	2,189	26 (0.82)	Not Reported	Not Reported	Vertebral

Ak et al. [2] examined 736 OC patients and found OM in 2.6%; OM was mostly seen with clear cell histology similar to our case report. The vertebrae, as in our patient, were most commonly involved, though patients often had multiple sites of involvement. Pain was the most common presenting symptom, but was absent in over 50%. Unlike our case, out of the two patients presenting with pathologic fractures, one had neurologic deficit. Patients were managed with palliative RT and bisphosphonates. Only advanced, inoperable disease at presentation was associated with a shorter time to development of OM. Median overall survival in OC patients with OM was 38.1 months and median survival after OM diagnosis was 13.6 months. Patients who had OM at the time of diagnosis of their OC had a shorter median OS than those who developed OM later on, 6.1 vs 63 months, respectively. To note, although insignificant, overall survival was shorter in patients with clear cell histology after OM than in patients with a different histologic subtype ( 7 vs 22 months) [2].

Sehouli et al. [5] examined 1,717 patients and found OM in 1.5%. The vertebral column was the most common site, most frequently in the lumbar, followed by the thoracic and cervical regions. Multiple OM were seen in the majority of patients, as in our case. Pain was the presenting symptom in 66%; 9% had impaired mobility unlike our patient, and 4% had neurologic symptoms. Pathologic fractures were reported in 33%. Most patients were treated with bisphosphonates; of those treated, 26% went on to develop pathological fractures and required surgical intervention. RT of OM was performed in a minority of cases for pain control and for SRE prevention. OM was noted to progress in 75% of cases despite systemic and local therapy. The median overall survival of the entire cohort was 50.5 months; patients with early OM had significantly shorter overall survival than those with later OM, 11.2 vs 78.4 months; to note, the overall survival rates were calculated regardless of the histology of the disease.

By far, the largest study on this topic, Zhang, C. et al. [6], examined 32,178 Surveillance, Epidemiology, and End Results (SEER) database OC patients and found the prevalence of OM was 1.09%. OM were more common in women over 65, Black and unmarried women. Similar to our patient, most of those patients had an advanced stage, poorly differentiated grade, non-serous type, elevated CA-125 and had concurrent distant metastasis. The median overall survival for the entire cohort was 50 months, whereas the median overall survival after OM diagnosis was 5 months only. Among examined variables, only surgery at the primary site was associated with significantly longer survival, 18 vs 3 months for primary site surgery versus no surgery. Survival was significantly shorter in patients with a non-serous histology without commenting specifically on the clear cell carcinoma histology [6].

Lastly, Zhang, M. et al. [7] found OM in 0.82% of 2,189 OC cases. The majority of OM were vertebral (12 cervical, 10 lumbar, seven thoracic), eight were pelvic, five were limb, two were sternal, and one was rib. Over half presented with pain, 35% with difficulty walking, and 15% were asymptomatic. Similar to our case report, the majority of cases with OM were in advanced disease and EOC, and most of them were managed with chemotherapy and RT. Cases that were managed with combined chemotherapy and RT had significantly longer survival than those treated with either agent alone, 14.2 versus 11 versus 8.4 months for combined therapy, RT alone, and chemotherapy alone, respectively [7].

There is a paucity of data regarding the management of OM with or without SRE in OC, and the current management of lesions is based on that for other solid tumors. Diagnosis includes history and physical, and imaging options include radiographs, CT and MRI; MRI is the modality of choice for vertebral lesions. A multidisciplinary team approach is often needed for the management of such rare cases, including radiology, orthopedic surgery, neurosurgery, radiation oncology , gynecological oncology, palliative and pain medicine [12]. All of these modalities were utilized as illustrated in this case.

## Conclusions

In conclusion, we present a unique case of clear cell EOC with vertebral OM resulting in pathologic C1 fracture requiring surgical stabilization, further complicated by vertebral artery encasement and narrowing. OM secondary to OC are rare and often present with pain though rarely with neurologic deficit. The risk factors for the development of OM are poorly understood but may include clear cell EOC as in this case, and lesions are more commonly described in those with late-stage disease. Patients who present with OM at the time of diagnosis or early in their disease course may have shorter overall survival than those with later OM. However, survival after OM diagnosis is on the order of months. Surgery at the primary site and combination chemotherapy and RT may prolong survival. These findings are based on limited retrospective studies, and further examination of risk factors and prognostic implications of OM in OC is needed.
